# Comparison of Testing Methods for the Detection of BRAF V600E Mutations in Malignant Melanoma: Pre-Approval Validation Study of the Companion Diagnostic Test for Vemurafenib

**DOI:** 10.1371/journal.pone.0053733

**Published:** 2013-01-10

**Authors:** Fernando Lopez-Rios, Barbara Angulo, Belen Gomez, Debbie Mair, Rebeca Martinez, Esther Conde, Felice Shieh, Jeffrey Vaks, Rachel Langland, H. Jeffrey Lawrence, David Gonzalez de Castro

**Affiliations:** 1 Laboratorio de Dianas Terapéuticas, Hospital Universitario Sanchinarro, Madrid, Spain; 2 The Royal Marsden NHS Trust and The Institute of Cancer Research, Sutton, London, United Kingdom; 3 Roche Molecular Systems, Pleasanton, California, United States of America; The Moffitt Cancer Center & Research Institute, United States of America

## Abstract

**Background:**

The cobas 4800 BRAF V600 Mutation Test is a CE-marked and FDA-approved in vitro diagnostic assay used to select patients with metastatic melanoma for treatment with the selective BRAF inhibitor vemurafenib. We describe the pre-approval validation of this test in two external laboratories.

**Methods:**

Melanoma specimens were tested for *BRAF* V600 mutations at two laboratories with the: cobas BRAF Mutation Test; ABI BRAF test; and bidirectional direct sequencing. Positive (PPA) and negative (NPA) percent agreements were determined between the cobas test and the other assays. Specimens with discordant results were tested with massively parallel pyrosequencing (454). DNA blends with 5% mutant alleles were tested to assess detection rates.

**Results:**

Invalid results were observed in 8/116 specimens (6·9%) with Sanger, 10/116 (8·6%) with ABI BRAF, and 0/232 (0%) with the cobas BRAF test. PPA was 97·7% for V600E mutation for the cobas BRAF test and Sanger, and NPA was 95·3%. For the cobas BRAF test and ABI BRAF, PPA was 71·9% and NPA 83·7%. For 16 cobas BRAF test-negative/ABI BRAF-positive specimens, 454 sequencing detected no codon 600 mutations in 12 and variant codon 600 mutations in four. For eight cobas BRAF test-positive/ABI BRAF-negative specimens, four were V600E and four V600K by 454 sequencing. Detection rates for 5% mutation blends were 100% for the cobas BRAF test, 33% for Sanger, and 21% for the ABI BRAF. Reproducibility of the cobas BRAF test was 111/116 (96%) between the two sites.

**Conclusions:**

It is feasible to evaluate potential companion diagnostic tests in external laboratories simultaneously to the pivotal clinical trial validation. The health authority approved assay had substantially better performance characteristics than the two other methods. The overall success of the cobas BRAF test is a proof of concept for future biomarker development.

## Introduction

The new paradigm of targeted drug development in cancer medicine is to design agents that inhibit specific recurring genetic lesions in tumors. A critical component of this model is the co-development of robust and accurate companion in vitro diagnostic (IVD) assays to detect these specific genetic lesions and thus to identify patients likely to benefit from a given targeted treatment [1].

**Figure 1:**
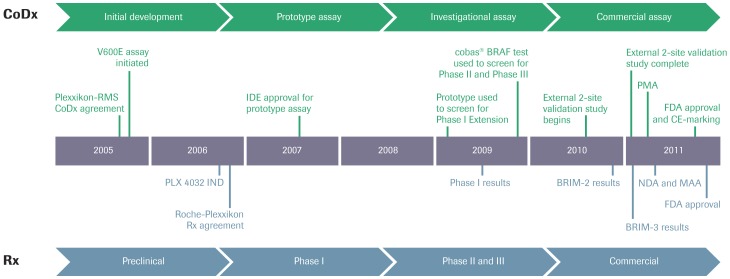
Key milestones in the co-development of vemurafenib and the cobas® 4800 BRAF V600 Mutation Test. Phases of companion diagnostic (CoDx) development in green, drug (Rx) development in blue. IDE = Investigational Device Exemption; IND = Investigational New Drug Application; MAA = Marketing Authorisation Application; NDA = New Drug Application; PMA = Premarket Approval Application; RMS = Roche Molecular Systems, Inc.

A successful example of this strategy is the focused and integrated co-development of the novel *BRAF* inhibitor vemurafenib and its companion IVD, the cobas 4800 BRAF V600 Mutation Test (“RT-PCR test”), which resulted in Food and Drug Administration (FDA) approval of vemurafenib in 2011, followed soon thereafter with CE-IVD marking in Europe - indicator that allows the free distribution of products within the European Union to meet essential requirements regarding safety, health and environmental protection-, less than 5 years after the Investigational New Drug Application (Figure 1) [2], [3]. Approval of vemurafenib was granted for the treatment of *BRAF* V600E mutation positive metastatic melanoma based on the results of a pivotal randomized phase 3 trial of vemurafenib *vs.* dacarbazine, which demonstrated that vemurafenib treatment results in significant improvements in overall survival, progression-free survival, and objective response rate [4]. The RT-PCR test, which was used to screen all patients enrolling on the trial, was approved at the same time to select patients for this therapy. Although the analytic performance of the RT-PCR test to Sanger sequencing has been compared at central laboratories using the positive RT-PCR specimens from the trials, there is no information on the reproducibility and performance of this assay using melanoma samples without prior knowledge of the *BRAF* mutation status [5].

This study compared the analytical performance of the CE-IVD marked and FDA-approved RT-PCR test with two other commercially available methods: bidirectional direct Sanger sequencing (“Sanger”) and the Applied Biosystems BRAF Mutation Analysis Reagents kit (“FA test”) for the detection of *BRAF* V600 mutations in formalin fixed paraffin embedded (FFPE) specimens of malignant melanoma. Specific objectives were to: (a) provide a realistic model for the pre-approval validation of companion diagnostic tests; (b) assess the frequency of invalid test results for each of the 3 methods; (c) compare three methods of *BRAF* V600E mutation detection, by calculating positive percent agreement (PPA) and negative percent agreement (NPA); (d) assess the reproducibility of the RT-PCR test at two independent laboratories; (e) assess the effects of tumor content, degree of pigmentation, and extent of necrosis on the analytical performance of the RT-PCR test; (f) evaluate the correct call rate for each method using DNA blends with 5% mutant alleles; (g) compare turnaround times between all methods.

## Methods

### Ethics Statement

The project has been approved by the institutional review board at Grupo Hospital de Madrid.

### Mutation Testing Methods

#### The cobas 4800 BRAF V600 Mutation Test kit

(“RT-PCR test”, Roche Molecular Systems, Inc., Branchburg, NJ, USA) is an FDA-approved and CE-IVD marked real-time PCR-based assay designed to detect the presence of the *BRAF* V600E (1799T>A) mutation in FFPE melanoma specimens. Full assay description and workflow is described in published manuscripts [5], [6]. Though designed to detect the V600E mutation, it has cross-reactivity with V600K, V600D and V600E2 (GTG>GAA) mutations.

#### The Applied Biosystems BRAF Mutation Analysis Reagents kit

(“FA test”, Applied Biosystems, Foster City, CA, USA) detects and differentiates three mutations in codon V600 of the *BRAF* gene (V600E, V600A, and V600G) using a shifted-termination assay primer-extension reaction and capillary electrophoresis fragment analysis [7].

#### PCR and 2× bidirectional direct Sanger sequencing

(“Sanger”) was performed to detect mutations in exon 15 of the *BRAF* gene following previously described protocols [8].

#### 454 sequencing

(GS FLX Titanium, 454 Life Sciences, Branford, CT, USA) [9] – a quantitative massively parallel pyrosequencing method – was performed using a validated protocol for *BRAF* mutation detection with a limit of detection (LOD) for V600E mutations of 1%. This method is a 5–7 day process that involves the generation of amplicons which are subject to pooling, ligation, emulsion PCR amplification, and massively parallel pyrosequencing. Data from this process is analyzed manually [9].

### Study Design

**Figure 2:**
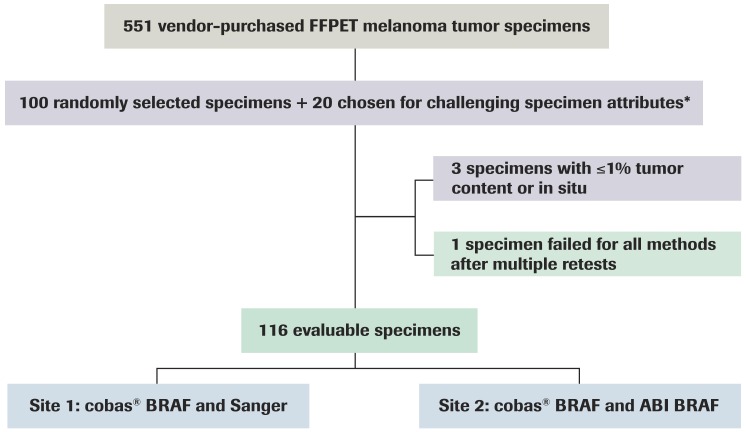
Study design and specimen selection. FFPET = formalin-fixed paraffin-embedded tissue. * Low tumor content (<50%); high levels of necrosis (≥50%); significant pigmentation (<10%); or non-V600E mutations.

The study was conducted using a blinded panel of FFPE tissue specimens of malignant melanoma as well as artificial DNA blends containing a low percentage of *BRAF* V600E mutant alleles (Figure 2). From a panel of 551 vendor-purchased specimens [Bioserve (Beltsville, MD), Cytomyx (Lexington, MA), Cureline (South San Francisco, CA) and Proteogenex (Culver City, CA)], 100 were selected at random, and an additional 20 were chosen for challenging attributes. Challenging attributes were defined as specimens with non-V600E mutations by Sanger sequencing, highly necrotic (as assessed by an external pathologist to be ≥50% necrotic), highly pigmented (as assessed by Roche Molecular using lab developed grading system protocol), and low tumor content (as assessed by an external pathologist as <50% tumor content by area).

Five 5 µm curls were sectioned from each of the 120 panel specimens and blinded. One section was mounted on a slide and stained with hematoxylin and eosin, coded, and reviewed by two pathologists (FL-R and EC). Each specimen was reviewed to confirm the diagnosis of melanoma, and to assess tumor content, degree of pigmentation, and extent of necrosis according to predefined criteria, which was based on laboratory experience in the study of somatic mutations in solid tumors [10]. Two curls per panel member were sent to Site 1 (Hospital Universitario Sanchinarro, Madrid, Spain) for analysis with the RT-PCR test and Sanger, and two were sent to Site 2 (The Institute of Cancer Research, Sutton, Surrey, UK) for analysis with the RT-PCR test and FA test.

DNA for the RT-PCR test was isolated from a single 5 µm section per panel member at each site using the cobas DNA Sample Preparation Kit. The DNA eluate was subsequently tested according to the package insert [11].

DNA for each of the other tests (Sanger and FA test) was isolated from a single 5 µm section per panel member using the QIAamp DNA FFPE tissue kit in the automated QIAcube system (Qiagen, Hilden Germany). The DNA eluate was then tested with Sanger according to a standard laboratory protocol or FA test according to the vendor-provided protocol.

Specimen retesting was permitted according to the manufacturer’s or procedure’s instructions as follows:

- RT-PCR test: <50% tumor content; insufficient DNA concentration; or invalid initial test result
- Sanger: no PCR amplification or difficult sequence interpretation
- FA test: fluorescence signal too strong; background noise; extra peaks that did not match any peaks from controls; or small mutation peaks that were difficult to identify as mutation signals.

454 sequencing was performed on all discordant specimens, all invalid specimens, and all specimens for which Sanger sequencing identified a non-V600E mutation.

### Invalid Test Rate

The number of invalid test results for the 120-member tumor panel was recorded and compared across the three testing methods.

### Methods Correlation

The positive percent agreement (PPA) and negative percent agreement (NPA) of the RT-PCR test for detecting *BRAF* V600E mutations with the other testing methods (Sanger and FA test) were evaluated. Discrepant analysis was performed with 454 testing of all specimens for which the RT-PCR test and comparison method gave discordant results and/or for which one of the two testing methods gave an invalid result. Test performance was characterized by comparing the evaluable paired results between RT-PCR test and Sanger, and between the RT-PCR test and FA test. The primary analysis focused only on the detection of V600E mutations as the RT-PCR test was designed for this indication. Additionally, the US licensed indication for vemurafenib is treatment of patients with V600E mutant disease. In cases where any method reported a different V600 mutant allele as ‘mutation detected’, this was reflected in the NPA findings. Calculations are defined in the statistical methods.

### Reproducibility

The reproducibility of the RT-PCR test was evaluated by comparing the results at the two independent clinical laboratory sites for each of the 120 FFPE panel members. Discrepant analysis was performed using 454 on all specimens with discordant results and/or an invalid result.

### Impact of Pathological Characteristics on Analytical Performance

The extent of pigmentation, necrosis, and tumor content in FFPE samples was graded, according to the following criteria, and their impact on the invalid test rate, mutation call rate, and reproducibility of the RT-PCR test was then assessed.

- Tumor content: high (≥50%) versus low (<50%)
- Tumor necrosis: high (≥50%) versus low (<50%)
- Pigmentation: high (≥10%) versus low (<10%).

### Correct Call Rate at Low Percentage Mutant Alleles

DNA blends with 5% V600E alleles (as determined by 454) were prepared from FFPE melanoma specimens. Twenty-four replicate blends (comprising 21 mutant-allele and three wild-type blends) were tested by respective pairs of methods at each site to assess the correct call rate at low percentage mutant alleles.

### Workflow Measures

Assay turnaround time from DNA isolation to results reporting was compared for all methods, assuming one 8-hour shift/day, and the following number of samples: 24 for RT-PCR test and Sanger, and 30 for FA test.

### Statistical Methods

For methods correlation, the two-sided 95% Wilson score confidence intervals were calculated for all measures of agreement [12]–[14]. The concordance study results will be summarized in tables of the form of Table 1 for each pair of comparisons.

**Table 1:** Example of Summary Table for Evaluation of Percent Agreement.

		Method 1		
		Positive	Negative	Total
Method 2	Positive	*a*	*c*	*a+c*
	Negative	*b*	*d*	*b+d*
	Total	*a+b*	*c+d*	*n*

In the table:

*a* = number of specimens tested positive by both Method 1 and Method 2.

*b* = number of specimens tested positive by Method 1 and negative by Method 2.

*c* = number of specimens tested negative by Method 1 and positive Method 2.

*d* = number of specimens tested negative by both Method 1 and Method 2.

The following statistics will be calculated:

•Overall Percent agreement between Methods = 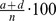.

•Positive Percent Agreement between Methods = 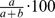.

•Negative Percent Agreement between Methods = 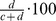.

95% confidence intervals for the above percent agreements will be calculated using methods described in CLSI EP12-A, User Protocol for Evaluation of Qualitative Test Performance; Approved Guideline, 2002.

False positive (FPR) and false negative (FNR) rates were calculated for both methods using the RT-PCR test as the reference using the formulae FPR = FP/(FP+TN) and FNR = FN/(TP+FN). TN and TP are observed numbers of true negatives and positives, and FP and FN are the observed numbers of the false positives and negatives, respectively.

For invalid rates, exact p-values for the differences between pairs of independent binomial proportions were calculated using commercial software (StatXact® v 9 by Cytel Software Corporation).

For correct call rate, the proportion of positive test results was compared. Minimum sample sizes were calculated that allow for the probabilities (power) 0.8, 0.9 and 0.95 to detect the difference between the proportions of positive test results between testing methods, along with the probability 0.05 of type 1 error of falsely rejecting the hypothesis of equivalence of the proportions. Asymptotic normal approximation of the distribution of the difference between pair of proportions was used. Exact p-values for the differences were calculated using commercial software (StatXact® v 9).

In all calculations, p-value ≤0.05 was considered statistically significant.

## Results

Of the 120 FFPE specimens, 116 were included in the method comparison analysis. Four samples were excluded due to insufficient tumor content/melanoma in situ (n = 3) or an invalid result with all three testing methods (n = 1) (Figure 2).

### Invalid Test Rate

Following initial testing and retesting (when necessary) according to manufacturer’s protocols, final invalid rates of 0%, 8·6%, and 6·9% were obtained for the RT-PCR test, FA test, and Sanger, respectively (Table 2). Differences between pairs of proportions 8/116–0/232 and 10/116–0/232 were statistically significant with exact p-values 1.2E-04 and 1.3E-05.

**Table 2:** Invalid test rates.

Assay	Initially invalid (n)	Invalid following retesting* (n)	Final invalid rate (%)
RT-PCR test (n = 232)	2†	0	0
Sanger (n = 116)	15	8	6·9
FA test (n = 116)	25	10	8·6

* Retesting was permitted according to the manufacturer’s or procedure’s instructions as follows: RT-PCR test: <50% tumour content, insufficient DNA concentration, or invalid initial test result; Sanger: no PCR amplification or difficult sequence interpretation; FA test: fluorescence signal too strong, background noise, extra peaks that did not match any peaks from controls, or small mutation peaks that were difficult to identify as mutation signals.

† The same sample was invalid when tested at the two sites.

### Methods Correlation with Sanger Sequencing

Of the 116 specimens tested at Site 1 using the RT-PCR test and Sanger, eight were invalid by Sanger, leaving 108 evaluable specimens for comparison. The initial agreement analysis showed PPA of 97·7%, NPA of 95·3%, and overall percent agreement (OPA) of 96·3% (Table 3). Following 454 sequencing, one specimen reported as ‘V600E’ by Sanger and ‘mutation not detected’ by the RT-PCR test was reported as ‘mutation not detected’ by 454. One specimen reported as ‘wild-type or non-V600E’ by Sanger and ‘mutation detected’ by the RT-PCR test was reported as V600E by 454. Two specimens reported as ‘V600K’ by Sanger and ‘mutation detected’ by the RT-PCR test were confirmed as ‘V600K’ by 454. Following discrepant resolution with 454 sequencing, the PPA was 100%, NPA was 96·9%, and OPA was 98·1% (Table 3). The RT-PCR test reported ‘mutation detected’ for all specimens confirmed to have V600E mutations by Sanger or 454, and ‘mutation not detected’ for all specimens confirmed to be mutation not detected or non-V600E by Sanger or 454. The FPR and FNR rates for Sanger were 2·3% and 4·7%, respectively, using RT-PCR test as the reference.

**Table 3:** Methods correlation between RT-PCR test and Sanger at Site 1 and after 454 sequencing of discrepant specimens.

N = 108		Sanger			Sanger and 454		
		V600E	Non-V600E/ wild-type	Total	V600E	Non-V600E/ wild-type	Total
**RT-PCR test**	**MD**	43	3*	46	44	2	46
	**MND**	1†	61	62	0	62	62
	**Total**	44	64	108	44	64	108
**Positive agreement**		97·7% (95% CI 88·2–99·6)			100% (95% CI 92·0–100)		
**Negative agreement**		95·3% (95% CI 87·1–98·4)			96·9% (95% CI 89·3–99·1)		
**Overall agreement**		96·3% (95% CI 90·9–98·6)			98·1% (95% CI 93·5–99·5)		

CI = confidence interval; MD = mutation detected; MND = mutation not detected.

* One sample subsequently confirmed as V600E by 454; Two samples subsequently confirmed as V600K;

† Subsequently confirmed as non-V600E/wild-type by 454.

### Methods Correlation with ABI BRAF Test

Of the 116 specimens tested at Site 2 using the RT-PCR test and FA test, ten were invalid by FA test, leaving 106 evaluable specimens for comparison. The initial agreement analysis showed PPA of 71·9%, NPA of 83·7%, and OPA of 77·4% (Table 4). The 24 specimens with discordant test results subsequently underwent 454 sequencing. Sixteen discordant specimens were reported as ‘V600E’ by FA test and ‘mutation not detected’ by the RT-PCR test. Twelve of these specimens were reported as ‘wild type’, three as ‘V600E2’ (GTG>GAA), and one as ‘V600R’ (GTG>AGG) by 454.

**Table 4:** Methods correlation between RT-PCR test and FA test sequencing at Site 2 and after 454 sequencing of discrepant specimens.

N = 106		FA test			FA test and 454		
		V600E	Non-V600E/ wild-type	Total	V600E	Non-V600E/ wild-type	Total
**RT-PCR test**	**MD**	41	8*	49	45	4	49
	**MND**	16†	41	57	0	57	57
	**Total**	57	49	106	45	61	106
**Positive agreement**		71·9% (95% CI 59·2–81·9)			100% (95% CI 92·0–100)		
**Negative agreement**		83·7% (95% CI 71·0–91·5)			93·4% (95% CI 84·3–97·4)		
**Overall agreement**		77·4% (95% CI 68·5–84·3)			96·2% (95% CI 90·7–98·5)		

CI = confidence interval; MD = mutation detected; MND = mutation not detected.

* Seven samples were reported as wild type by FA test and ‘mutation detected’ by RT-PCR test of which four were subsequently found to be V600E by 454, and three to be V600K. One sample was reported as V600G by FA test and ‘mutation detected’ by the RT-PCR test and was subsequently found to be V600K by 454.

† Twelve samples subsequently reported as wild type, three as V600E2, and one as V600R by 454.

Of the remaining eight discordant specimens, seven specimens were reported as ‘wild type’ by FA test and ‘mutation detected’ by the RT-PCR test. Four of these seven were reported as ‘V600E’ and three as ‘V600K’ by 454. One discordant specimen reported as ‘V600G’ (GTG>GGG) by FA test and ‘mutation detected’ by RT-PCR test was reported as ‘V600K’ by 454. Following discrepant resolution with 454 sequencing, the PPA was 100%, NPA was 93·4%, and OPA was 96·2% (Table 4). The FPR and FNR rates for FA test were 28% and 16%, respectively, using RT-PCR test as the reference.

Consistent with the results observed at Site 1, the RT-PCR test gave a ‘mutation detected’ result for all specimens confirmed to have V600E mutations by Sanger or 454, and a ‘mutation not detected’ result for all specimens confirmed to be wild-type or non-V600E by Sanger or 454.

PPA and NPA between RT-PCR test and Sanger are statistically significantly higher than those between RT-PCR test and FA test based on the non-overlapping 83% confidence intervals for the respective percent agreement estimates [14].

### Reproducibility

Of the 116 specimens evaluable at each site using the RT-PCR test, 95·7% produced concordant results. The agreement for V600E mutation-positive specimens was 100%, and the agreement between the wild-type specimens was also 100% as determined by Sanger or 454. The remaining five discordant specimens between sites were V600K mutation-positive by 454 sequencing.

### Impact of Pathological Characteristics on Analytical Performance

Pathological assessment of the 116 FFPE specimens revealed varying degrees of pigmentation, necrotic tissue, and tumor content (Table 5). Although we believe that none of these pathological characteristics had an observable effect on the invalid rate, reproducibility of the RT-PCR test between sites, or agreement analysis with Sanger or FA test, 29% and 65% of the samples were unclassifiable for their pigmentation and necrosis characteristics, respectively (Table 5). Therefore, a formal statistical analysis was not performed. The only fact worth mentioning was that one specimen that was initially invalid by the RT-PCR test was highly pigmented; however, it gave a valid result when retested as recommended in the RT-PCR test package insert [10].

**Table 5:** Distribution of pathological characteristics.

Characteristic	Unclassifiable	Low	High
Pigmentation^a^	34	27	55
Necrosis^b^	75	23	18
Tumour content^b^	NA	36	80

NA = Not applicable.

a Low = <10%; high = ≥10%.

b Low = <50%; high = ≥50%.

### Correct Call Rate

Correct call rate at low mutant alleles for V600E was assessed for each method using 24 replicates of a 5% mutant allele DNA blend. The correct call rate for the RT-PCR test was 100% (48/48 samples) *vs.* 33% (8/24 samples) for Sanger and 21% (5/24 samples) for FA test. Differences between pairs of proportions were statistically significant with p-values 1.0E-06 for both methods. Thus the proportions of correct calls were statistically significantly higher for the RT-PCR test compared to Sanger and FA test.

### Workflow Measures

Turnaround time was ∼1 day for the RT-PCR test, ∼5 days for Sanger, and ∼2 days for FA test.

## Discussion

Although the RT-PCR test is CE-IVD marked and is currently the only FDA approved test for the identification of patients with the V600E mutation, a number of assays are widely used in clinical practice [15], [16]. However, these techniques have not been systematically compared and it is not clear which test provides the best performance. It is also unclear which of the currently available methods is best for testing FFPE clinical material, and can achieve acceptable turnaround times for clinical decision-making. We sought to provide these data and share the analytical validation that was performed simultaneously to the pivotal clinical trial.

The RT-PCR test detected 100% of V600E mutations and 100% of wild-type specimens in the FFPE panel and had the lowest invalid rate of the three methods. Although pigmentation had an initial effect on one of the FFPE specimens tested, after retesting according to the manufacturer’s recommendations, RT-PCR test result on this specimen was valid, resulting in a 100% valid test rate overall. Obtaining invalid tests results has unfavorable implications for patients, as the need to repeat tests and potentially to re-biopsy the patients can lead to significant delays in patients receiving treatment.

Test reproducibility between different laboratories and different users is another key attribute of an IVD. In a previous study at 3 external sites (2 in the United Stated and 1 in Australia), we had observed an overall reproducibility of 98·8% for the RT-PCR test, but only based on an 8-member panel of melanoma samples [5]. In this study, we observed an overall reproducibility of 95·7%. This variance in reproducibility provides further evidence for the validity of our approach (i.e., external validation outside pivotal clinical trials). The reproducibility of the RT-PCR test for reporting V600E (1799T>A) mutations and wild-type cases was 100%, and the five discordant specimens all harbored V600K mutations, as demonstrated by Sanger and 454 sequencing.

Although designed to detect the V600E mutation, the RT-PCR test also has cross-reactivity with non-V600E mutations. Preclinical studies show that cell lines harboring V600K, V600D, and V600E2 mutations are sensitive to vemurafenib, and limited clinical data suggest that patients with V600K-mutant melanomas may respond to targeted therapy [17]–[19]. In the BRIM2/3 clinical trials, V600K accounted for ∼12% of mutations (V600E accounted for ∼82% of mutations), and RT-PCR test detected 70% of V600K in this cohort [5]. Following this line of reasoning, there are several questions that will need to be addressed in the future. First, should we detect all clinically relevant *BRAF* mutations or just the common ones?. Second, do we need comprehensive detection of the codon 600 variants in the *BRAF* mutant population?. Third, what is the cost-effectiveness of sequencing wild-type cases to identify the non-V600E mutants that the RT-PCR assay might miss?.

The differences in the published LOD for the three test methodologies were clearly highlighted when testing artificial tumor blends with 5% mutant *BRAF* alleles. The detection rate of the RT-PCR test was considerably higher than that of the other two methods. The low detection rate for Sanger sequencing (33%) was consistent with previously published studies, which indicated that the LOD for Sanger sequencing was 25–50% [15]. The low detection rate of 21% for the FA test observed in this study was surprising as the data for this test suggest that it detects 1–5% mutant to wild-type genomic DNA [7]. It must be emphasized that our LOD studies were performed using FFPE specimens (i.e., identical to the clinical reality). Highly sensitive methods of mutation testing are particularly important given the recently reported intra- and inter-tumor heterogeneity of *BRAF* V600E mutations in melanoma [20].

Finally, one practical comment is important as more such companion diagnostic assays are developed. Low levels of reimbursement may influence the choice of methodology (Sanger being the cheapest option and the RT-PCR assay the most expensive assay of the three presented herein). However, it must be emphasized that costs vary tremendously between molecular diagnostic laboratories due to a number of reasons: fully automated DNA extraction *versus* manual extraction, mutation testing in batches *versus* case-by case testing, cost of interpretation time and reporting, ease of implementation, etc….

Nevertheless, the cost of a specific test should take into account its performance (i.e., invalid rate, LOD and concordance with the gold standard).

In summary, we have presented a comparison study of three different methods for the detection of V600E mutations in the *BRAF* gene in FFPE specimens of malignant melanoma. The findings add support to the value of using extensively validated health authority approved tests. It is feasible to evaluate potential companion diagnostics in external laboratories simultaneously to the pivotal clinical trial validation (Figure 1). The overall success of the cobas 4800 BRAF V600 Mutation Test (RT-PCR test) is a proof of concept for future biomarker development.
